# Early Check: translational science at the intersection of public health and newborn screening

**DOI:** 10.1186/s12887-019-1606-4

**Published:** 2019-07-17

**Authors:** Donald B. Bailey, Lisa M. Gehtland, Megan A. Lewis, Holly Peay, Melissa Raspa, Scott M. Shone, Jennifer L. Taylor, Anne C. Wheeler, Michael Cotten, Nancy M. P. King, Cynthia M. Powell, Barbara Biesecker, Christine E. Bishop, Beth Lincoln Boyea, Martin Duparc, Blake A. Harper, Alex R. Kemper, Stacey N. Lee, Rebecca Moultrie, Katherine C. Okoniewski, Ryan S. Paquin, Denise Pettit, Katherine Ackerman Porter, Scott J. Zimmerman

**Affiliations:** 1 Center for Newborn Screening, Ethics, and Disability Studies, RTI International, 3040 E. Cornwallis Rd., Research Triangle Park, NC 27709 USA; 2 RTI International, Seattle, WA USA; 3 Duke University, Durham, NC USA; 4 Wake Forest School of Medicine, Winston-Salem, NC USA; 5 University of North Carolina at Chapel Hill School of Medicine, Chapel Hill, NC USA; 6 RTI International, Washington, DC USA; 7 Nationwide Children’s Hospital, Cincinnati, OH USA; 8 RTI International, Chesterfield, MO USA; 9 RTI International, Minneapolis, MN USA; 10 North Carolina State Laboratory of Public Health, Raleigh, NC USA

**Keywords:** Newborn screening, Rare disorders, Translational science

## Abstract

**Background:**

Newborn screening (NBS) occupies a unique space at the intersection of translational science and public health. As the only truly population-based public health program in the United States, NBS offers the promise of making the successes of translational medicine available to every infant with a rare disorder that is difficult to diagnose clinically, but for which strong evidence indicates that presymptomatic treatment will substantially improve outcomes. Realistic NBS policy requires data, but rare disorders face a special challenge: Screening cannot be done without supportive data, but adequate data cannot be collected in the absence of large-scale screening. The magnitude and scale of research to provide this expanse of data require working with public health programs, but most do not have the resources or mandate to conduct research.

**Methods:**

To address this gap, we have established Early Check, a research program in partnership with a state NBS program. Early Check provides the infrastructure needed to identify conditions for which there have been significant advances in treatment potential, but require a large-scale, population-based study to test benefits and risks, demonstrate feasibility, and inform NBS policy.

**Discussion:**

Our goal is to prove the benefits of a program that can, when compared with current models, accelerate understanding of diseases and treatments, reduce the time needed to consider inclusion of appropriate conditions in the standard NBS panel, and accelerate future research on new NBS conditions, including clinical trials for investigational interventions.

**Trial registration:**

Clinicaltrials.gov registration #NCT03655223. Registered on August 31, 2018.

## Background

Newborn screening (NBS) is a public health program established to identify and treat babies with life-threatening or debilitating disorders before clinical symptoms appear. Determining which of the hundreds of known childhood-onset disorders should be included in NBS is a complex public health challenge in which major and sometimes competing perspectives (e.g., medicine, science, public policy, advocacy, ethics, economics, politics) converge [1]. The evidence base available to inform decisions about which conditions should be included in NBS is often limited, making it difficult to anticipate with confidence the full range of outcomes that may occur if screening for a disorder were adopted [2]. Policy makers must often rely on incomplete data to evaluate competing claims of those who advocate for a rapid expansion of the NBS panel versus those who advocate for a more deliberative pace [3].

To address the substantial gaps in NBS evidence, we have established Early Check, a research enterprise embedded in a public health program to inform NBS policy. Early Check addresses a fundamental translational science conundrum—how to quantify the potential benefits and risks of early identification and presymptomatic treatment for infants who have rare disorders—by building a statewide system in which testing for a select number of conditions is offered as a supplement to standard NBS in North Carolina (NC) to all birthing parents under a voluntary research protocol. The complex challenges in building such a program have required innovative solutions and collaboration among multiple stakeholders.

## Newborn screening policy considerations

NBS programs around the world vary widely in the number of disorders screened [4], but the core decision-making criteria are relatively standard: (a) the condition is a significant health problem; (b) its natural history is well understood; (c) there is an affordable and accurate screening test; (d) proven treatments exist that are more effective if initiated earlier than is possible with usual clinical case detection; and (e) governmental entities are capable of screening and providing the necessary follow-up [5].

Timing and urgency of treatment are central to NBS policy. The first few postnatal weeks are viewed as a critical period, a window of time in which treatment and/or surveillance must begin to maximize benefit and minimize adverse health outcomes for certain conditions [6]. The fact that symptoms are not obvious at birth, combined with the need for rapid detection and treatment, means that NBS identifies infants in a possible state of “medical emergency” [7]. Urgency of treatment is the primary justification for population screening, generally under an opt-out model (parents receive information and screening is conducted unless they explicitly reject it). Although the role of parent choice in NBS has been debated (e.g., [8, 9]), the opt-out approach is generally well accepted across the United States, with very few parents refusing screening [10]. But because NBS is a universal public health program, policy makers must have a high degree of confidence, with supporting evidence, that screening the entire population is warranted.

## Challenges and gaps in evidence for NBS policy

The typical review of NBS candidate conditions includes clinical considerations, research findings, policy implications, and input from advocacy groups or other stakeholders [11]. Ideally, screening decisions should rest heavily on scientific evidence but, as with most rare disease research, the available data are seldom fully adequate [12], with several notable gaps.

### Understanding prevalence and natural history

One of the most common challenges is that the true prevalence, full clinical spectrum, and natural history of a disorder are often unknown [13]. Prospective natural history studies typically begin after a diagnosis is made, and thus understanding prediagnostic natural history relies on parent recall, review of medical records, or observations of disease progression in siblings. These methods are flawed in that they are biased toward identified patients. Individuals from low-income, ethnic minority, or other underserved groups may be inadequately represented, and milder cases or those with atypical late-onset manifestations are less well known. Population screening is the only way to determine the true prevalence of a disorder and understand the full relationship between biomarkers and disease expression [14].

### Inadequate treatment data

A second common evidence gap is limited data on potential therapies or interventions. Initial trials of promising treatments often are conducted with children who already exhibit symptoms, raising the risk of trial failure for a drug that may have been effective if it had been delivered before symptoms appeared. Even when benefit is demonstrated, treatment trials may not yield evidence of a critical presymptomatic period during which treatment may be most effective. Trials including presymptomatic infants often are limited to samples of convenience (e.g., siblings of affected children), and may have insufficient power to detect variability in response, especially for a disorder that varies in terms of severity and age of onset [15]. Furthermore, policy decisions are often made on short-term efficacy data, lacking important information about long-term outcomes for children and families [16, 17].

### Small samples

To conduct natural history studies with representative populations or clinical trials of presymptomatic treatments of infants with rare disorders, a study would need to screen hundreds of thousands of babies shortly after birth, an enormous undertaking. Historically, such studies have recruited participants using: (1) presymptomatic screening offered with parent permission in a network of hospitals; (2) identification of young children with very early stage disease through specialty clinics; or (3) recruitment of younger siblings of affected children through clinics, advocacy groups, or online. Even taken together, these recruitment strategies will fail to acquire an adequately sized study sample. The need to obtain informed consent from thousands of families to conduct screening, natural history, or treatment studies remains a formidable barrier to NBS research.

### Building and rebuilding research networks

Most NBS studies focus on a single disorder and are led by investigators with disease-specific expertise. Funding typically comes from a governmental agency (if the disorder aligns with their priorities), a pharmaceutical company (if it has a treatment for the disorder that needs testing), or a patient-advocacy group (if the study addresses their disorder). Focusing on a single disorder increases the ability to attract research funds, but teams conducting these projects may not be interested in other disorders, and funders are not interested in supporting projects for conditions that do not fit their mission. When the project is completed, the infrastructure is usually dismantled, leaving teams interested in other disorders to start anew.

## Methods/design

We are building an infrastructure and assessing the outcomes of a statewide research enterprise embedded in a state public health program to address these problems, offering voluntary testing for a selected number of conditions under a research protocol in NC, a U.S. state with a diverse population (56% white non-Hispanic, 24% African American, 16% Hispanic, 4% other) and more than 120,000 births per year. The initial Early Check launch offers screening to detect two conditions: spinal muscular atrophy (SMA) and fragile X syndrome (FXS). We anticipate that, as the evidence base supporting the safety and efficacy of screening for these conditions builds, they may be removed from the Early Check study panel and other conditions will be added, providing an infrastructure for continuous NBS research.

**Fig. 1:**
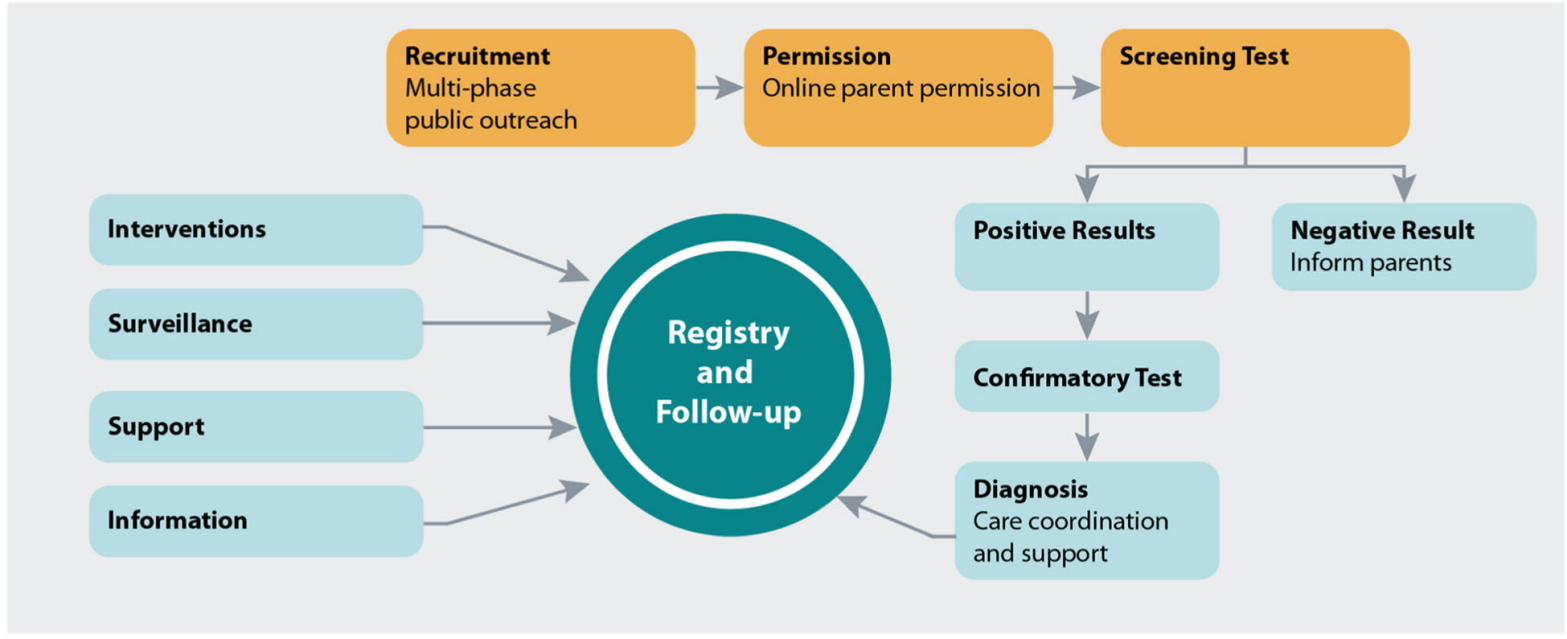
Early Check Protocol

Figure 1 illustrates how Early Check works. In brief, a variety of strategies have been developed and tested for outreach and recruitment, each of which provides a link to an online electronic permissions module describing the study and providing decision support resources. After the mother of a newborn has given permission, Early Check laboratory scientists conduct testing for the specified conditions using residual dried blood spots collected for standard NBS. Parents of newborns who screen negative for Early Check conditions receive their results via an online portal. Families with infants identified as being at risk for an Early Check disorder (screen-positive) are referred to the clinical research team. A genetic counselor on the research team calls and explains screen-positive results, provides counseling, and coordinates diagnostic confirmation.

Long-term follow-up and clinical care vary by condition and are driven by family preference and project resources. Parents of screen-positive infants are invited to participate and allow their child to participate in a longitudinal registry and research-related developmental follow-up. The project provides parents with information about potential treatment options to support families who are considering participation in clinical trials. Follow-up assessments of family and child outcomes examine how well the family adapts to Early Check screening and diagnostic information, and whether and why parents decide to participate in a clinical trial. A range of evaluation strategies assesses use patterns and parent perceptions of the program.

## Key components of Early Check

Early Check is a deceptively simple concept: Choose some critical conditions for which evidence is needed, offer screening of newborns to all new parents, and conduct follow-up studies to determine potential benefits and risks of harm. However, the design and implementation of a research program in partnership with a state public health program has proven to be a complex endeavor, requiring teams of investigators and multiple partners to develop and test solutions. Key activities have included building a coalition of investigators, stakeholders, and funders; selecting disorders to be screened; designing, implementing and evaluating statewide outreach and recruitment strategies; developing a web portal and electronic permission/consent model; establishing robust and secure laboratory processes; building a comprehensive follow-up program; and evaluating outcomes.

### Building a coalition of investigators, stakeholders, and funders

Early Check is an interdisciplinary and multi-institutional collaboration, led by investigators in the Center for Newborn Screening, Ethics, and Disability Studies and colleagues in the Center for Communication Science at RTI International (a nonprofit research institute), working in partnership with faculty at the University of North Carolina at Chapel Hill School of Medicine (UNC-CH) (clinical follow-up); Wake Forest School of Medicine (ethics and follow-up); Duke University (laboratory and clinical trial consultation); and the North Carolina Division of Public Health (NC-DPH) and the NBS program located in the North Carolina State Laboratory of Public Health (NCSLPH) (initial outreach to families and access to residual dried blood spots). The Office of Human Research Ethics at UNC-CH serves as the central Institutional Review Board (IRB), and each partner institution has agreed to rely on its review.

Essential to the success of Early Check is our partnership with the NCSLPH, necessitating a trusting relationship that respects and protects the public health mission of the state’s NBS program while establishing a rigorous research enterprise to support the program’s core mission. Prior to Early Check, RTI began a collaboration with NCSLPH by hiring staff and securing funds to conduct pilot studies and purchase equipment for several conditions already recommended for screening (but not yet implemented in NC). Success with these activities led to a Business Associate Agreement (BAA) between RTI and the NCSLPH, allowing us to share protected health information between entities. This agreement enabled recruitment of all infants in NC and provided a mechanism for matching parent permissions with their child’s dried blood spot, eliminating the need for an office visit or extra blood draw.

### Selecting disorders to be screened

Early Check is designed to assess the benefits of screening for disorders that are not currently part of the NBS panel. The optimal disorder for Early Check is one for which significant advances in treatment potential have been made, but for which a large-scale, population-based study is still needed to realistically estimate possible benefits and risks of harm, demonstrate feasibility, and inform policy. Five criteria common to standard NBS guide the first step in decision making. The condition must (1) result in serious problems in health or development and have subsequent adverse impacts on families; (2) have significant symptoms that emerge in the first 3 years of life; (3) be difficult to diagnose early in the disease process; (4) be detectable through a low-cost, accurate, and feasible screening test that can be performed on residual dried blood spots; and (5) have follow-up guidelines or protocols which have at least moderate certainty of providing net benefit for the child and family.

Conditions meeting these criteria are then subject to project-specific considerations: (1) the disorder must be sufficiently common to answer predetermined research or policy questions within a reasonable period of time; (2) the condition has potential treatments (medical or behavioral) that need systematic study to determine presymptomatic benefit; (3) patient advocacy groups endorse research to test the benefits of earlier identification; and (4) funds are available to support condition-specific costs for screening and follow-up.

Following a review of numerous conditions, we selected FXS and SMA for the initial launch because (a) they exemplify a wide range of medical consequences, treatment urgency, treatment approaches, and consequences for families; (b) we have substantial clinical and research expertise with each, including a prior pilot NBS study for FXS [18] and significant experience with SMA screening [19, 20] and treatment; (c) important research questions remain that need to be addressed; and (d) we were able to obtain additional funding and contributions necessary for screening. We also offer parents of participating infants the option of receiving FXS premutation results under a substudy, with a separate consent process. These two disorders serve as the initial prototypes to implement the program, but our envisioned future includes an expanded set of disorders. Early Check does not currently provide the option for mothers to choose among the disorders offered, as doing so would substantially complicate our permission process and screening flow. However, as we expand the panel and establish our infrastructure, a future possibility would be to explore innovative ways to seek permission for an individualized set of disorders.

### Designing, implementing, and evaluating statewide outreach and recruitment strategies

Failure to recruit enough participants for clinical trials is a significant barrier to developing and evaluating new therapies [21]. This problem is well documented, and several reviews have examined strategies to improve enrollment and retention [22, 23]. Recruitment of minority and low-income participants is a problem of special concern [24]. Most clinical trial recruitment is conducted with patients who have a known illness or disorder (e.g., [25]), often through clinics, patient registries, or electronic medical records, none of which were practical for Early Check. The project has required our outreach team to address three important challenges: (1) informing every new family in NC about the opportunity to participate; (2) distinguishing Early Check from standard NBS; and (3) getting parents to visit the website to consider study participation, even when their infant seems healthy.

Ideally, in-person recruitment would be used [18, 26], but in a state with over 120,000 births per year in more than 100 hospitals, in-person recruitment is impractical and prohibitively expensive. Our recent survey found that, generally, women are interested in participating in programs like Early Check [27], so a key activity has been testing and evaluating remote recruitment procedures to reach these women and their families. An analysis of outreach and recruitment approaches in the National Children’s Study found that there is no magic bullet for outreach and recruitment, and multiple strategies are required [28]. We designed a systematic, evidence-based, multiphase method approach for large-scale recruitment that excludes in-person recruitment (at least for the present time).

To develop effective and motivating outreach materials, we engaged in extensive formative evaluation including literature reviews, 26 focus groups, and online experiments, the most recent conducted with 200 women representing our target population. We tested the acceptability of sending letters, e-mails, text messages, and preferences for graphics and ad concepts. Focus groups included English and Spanish speakers, as well as community members from Lumbee and Eastern Cherokee groups. The experiences shared by focus group participants allowed us to identify important barriers and facilitators to enrolling. We then conducted an online experiment comparing various messages and graphical elements against each other to determine the most effective ad elements that need to be displayed to motivate visits to the Early Check website (www.earlycheck.org). Reading-level analyses of web content indicated that most sections are at Grade 8 or lower, meeting or exceeding recommended standards for comprehension.

To systematically evaluate the effectiveness of various outreach strategies, we are using a phased approach spanning both prenatal and postnatal periods. Each phase builds on the previous one, allowing us to evaluate the additive value of new strategies using assessments of both process (documentation of the outreach activities) and outcome (how many parents visit the Early Check website and electronic permission portal). We will use interrupted time series analyses to assess the impact of outreach strategies on visits to the permission portal and ultimately on uptake rates.

- Phase I focuses on the postnatal period, the only time during which we can reach almost every family in the state. During this phase there are three components: (a) a letter on NC-DPH letterhead and an Early Check flyer mailed within 5 days after birth; (b) an e-mail that communicates the same information as the letter and flyer to parents for whom an e-mail address is available; and (c) outreach to providers and professional associations to build awareness and reinforce a single message: *If parents ask about Early Check, encourage them to visit the electronic permissions portal to learn more and enroll if they are interested*. Phase I will continue for approximately 6 months or until a stable enrollment baseline is achieved. As part of Phase I we are embedding an experiment in selected counties to understand if reminder cards can increase visits to the Early Check website and permission rates.
- Phase II adds a social media component to Phase I activities, targeting both prenatal and postnatal women. Research on the effectiveness of social media recruiting is variable, but generally shows that social media advertising, such as through Facebook, can increase study recruitment, sometimes significantly, at a relatively low cost [29–31]. During this phase, we will embed Early Check ads and relevant content in social media platforms used by pregnant women and those who have recently had a baby. A comprehensive and multifaceted strategy will leverage partners and influencers to amplify social media messaging, seeking earned media opportunities, and integrating social media into the Early Check website. Phase II will continue for up to 6 months to determine whether social media messaging results in a significant increase in visits to the permissions portal beyond Phase I. To understand if we can increase engagement in our social media strategy as well as visits to the Early Check website, we will manipulate aspects of ads promoting the project in social media feeds to determine if some requests to join the study are more motivating than others.
- As we assess the results from Phases I and II, we will evaluate other strategies to add to subsequent phases, either statewide or in the form of experiments by region, hospital system, or target population. We are developing protocols to recruit pregnant women by posting messages about the study on their patient portals and formulating a range of strategies to inform women about Early Check in health care provider offices, birthing classes, and hospitals. Given NC’s geographic diversity and population distribution, we will consider specialized strategies for accessing and building trust among “hardly reached” groups—individuals who are traditionally underrepresented in research studies [32]—such as providing information to low-income mothers participating in the federally sponsored Special Supplemental Nutrition Program for Women, Infants, and Children. In-person recruitment will likely be necessary in selected hospitals.

Our ultimate goal is to identify the combination of approaches most likely to maximize the number of visits to the Early Check website to consider enrolling in the project. Throughout, we emphasize the difference between Early Check and standard NBS, because research suggests that parents are more likely to consider participation in an optional research study when information about the study is presented in the context of regular NBS [33].

### Developing a web portal and electronic permission/consent model

The main goal of outreach is to send mothers to the electronic permission portal to consider enrolling. Appreciation for the potential of electronic consent and the inclusion of multimedia content has grown considerably in the last few years. This is due in part to anticipated regulatory changes as well as the potential superiority of e-consent approaches to tailor content and use pictures and videos to more clearly describe a study [34, 35]. An electronic permission process is the only feasible way we could enroll mothers on such a large scale.

Our goal was to create a feasible, ethical, and reproducible approach to large-scale informed choice. To accomplish this, we designed a permissions module with a responsive user-oriented design process integrating formative evaluation and ongoing quality improvement activities. The module (a) meets regulatory requirements for informed consent/parental permission; (b) differentiates Early Check activities from standard NBS; (c) uses plain language to explain concepts; (d) minimizes time burden; (e) uses graphics, voiceover, and whiteboard videos to enhance interest and clarify information; (f) includes decision support to help mothers decide whether study participation is right for them; and (g) provides access in both English and Spanish. A reading level analysis of the permissions portal indicated an overall average reading grade level of 6.

The decision support component presents reasons mothers may want to enroll their infant (no cost, no additional blood draw required, information of potential use to families, potential benefits to infants) as well as reasons they might not want to enroll (increased anxiety about their child’s health or worry about privacy and data security). Mothers who have additional questions can e-mail, call, or participate in a chat session with research staff. Although we want to recruit as many families as possible, we provide a balanced perspective and emphasize informed choice.

Formative interviews with mothers of newborns were used to evaluate the permissions text and determine whether women felt sufficiently informed. We observed participant interactions with the portal using a minimally prompted, think-aloud approach to identify any problems related to content, such as concepts that were difficult to comprehend, desired additional information, as well as any navigational or functional enhancements needed. In the first phase of Early Check implementation, we use data from Google Analytics, a service that tracks and reports website traffic, to evaluate the permissions model by examining variables such as length of time interacting with the educational components in the module and where parents are most likely to stop engagement. We will evaluate the permission conversion rate (i.e., the percent of unique users who give permission out of all who initiate use of the permission portal) and will assess sociodemographic factors associated with providing or declining permission for screening. If mothers do not enroll, we ask a brief optional question regarding reasons for not joining. Finally, we will evaluate the Early Check enrollment experience and information recall using interviews and surveys. Longer-term, we will use the results from this first set of analyses to develop and systematically evaluate alternative approaches and/or modalities for providing key content.

### Establishing robust and secure laboratory processes

Our partnership with NCSLPH has required careful attention to many details, including access to protected health information, use of residual dried blood spots, accurate and valid laboratory screening procedures, and secure information management systems. Each day, 300–400 infants are born in NC; blood spots are collected in the birthing hospital and demographic data are entered on the NBS card, which is shipped to the NCSLPH. Under our BAA, the NCSLPH exports demographic data daily to RTI’s permission matching data base, which resides in our secure Federal Information Process Standard (FIPS) moderate environment. This environment is held to a high security standard and is located in a segregated network area inside the RTI firewall, to ensure that data access is restricted to authorized team members. Two-factor authentication and specific Health Insurance Portability and Accountability Act (HIPAA) training are required to access the FIPS moderate environment. The data are matched with the enrollment data from the electronic permissions portal, after which research staff access the residual blood spot for each infant for whom permission is provided.

A primary criterion for Early Check is the existence of an accurate screening method that is affordable and can be implemented in a high-throughput public health environment. Our research team evaluates various screening methods and procures equipment that is not available in the NC NBS program. The Early Check laboratory is certified by the Centers for Medicare & Medicaid Services under the Clinical Laboratory Improvement Amendments (CLIA). The research team has established a rigorous validation and quality control protocol. Working under a CLIA director, research staff conduct screening for the specified conditions after all protocols have been established and validated.

As we consider adding new conditions to the Early Check panel, our team will explore other technologies that are accurate and feasible for high throughput NBS (e.g., [36]). We aim to incorporate additional tests to address the growing expectation that NBS should identify molecular variations to better characterize a disorder. The possible incorporation of whole exome or genome sequencing into NBS [37] has been under considerable debate, but this method is expensive, time-consuming, and produces many results that do not fit NBS criteria, and is currently premature for high volume testing [38, 39]. Thus, genetic testing in NBS currently focuses on testing to detect specific pathogenic variants or sequencing single genes as a second-tier screen to reduce false-positive results. Early Check currently uses real-time quantitative polymerase chain reaction (qPCR) methods for SMA and PCR combined with capillary electrophoresis for FXS screening; however, future disorders may require methods such as droplet digital PCR (ddPCR), a next-generation PCR method that is more sensitive, precise, and reproducible than conventional PCR when quantifying nucleic acids [40] and determining copy number variants [41].

### Building a comprehensive follow-up program

For infants who screen positive for an Early Check condition, we have built a comprehensive follow-up program to (a) determine the accuracy of our screening through diagnostic confirmation; (b) refer infants who are confirmed to have the condition for specialty care and treatment; (c) maximize support for families as they learn about their child’s condition and navigate decisions about treatment and study options; (d) enable research on treatment efficacy; (e) enroll infants in longitudinal natural history studies; (f) study family adaptation to their child’s condition; and (g) evaluate the usefulness of information families receive. Accomplishing these activities has required approaches that are developed specifically for each disorder. Both process and outcome evaluation activities will study the acceptability and usefulness of the information, support, and services provided, as well as the long-term outcomes experienced by children and families.

Mothers of infants with screen-negative results receive an e-mail informing them that they can sign in to their user account on the electronic permissions portal, where they will find a screen-negative report and a lay summary of the result. For screen-positive babies, a certified genetic counselor on the research team contacts the family via phone within 24 h of receiving the screen-positive result. Confirmatory testing procedures are then offered in collaboration with clinical partners at UNC-CH. Some conditions, like SMA, require a blood draw; for others, like FXS, confirmatory testing can be done by mailing cheek swab kits for saliva testing. Confirmatory testing is conducted in a CLIA-certified diagnostic laboratory. Parents are contacted by the genetic counselor on the research team as soon as confirmatory test results are available. For those with negative (normal) confirmatory testing, families are offered the opportunity to participate in a phone or in-person genetic counseling session to understand the false-positive result. A letter is sent with a copy of the normal lab report. Those with a confirmed diagnosis have the option to receive genetic counseling, a medical and developmental evaluation, and referrals for appropriate treatment, the specific protocols of which vary considerably by disorder. All infants are referred to appropriate specialists, such as neurology, pulmonology, feeding/nutrition, early intervention, and developmental surveillance for ongoing clinical care.

Following diagnostic confirmation and referral to specialty services, families are given the opportunity to attend a newly formed Early Check specialty clinic established at UNC-CH. This clinic provides interdisciplinary evaluations, consultation, and treatment recommendations, and is available on an ongoing basis to all families of children with confirmed diagnoses regardless of whether they participate in follow-up research studies. Families are also invited to participate in a longitudinal registry. The registry and associated follow-up activities are designed to provide ongoing support for families—supplementing, rather than replacing, the supports and services provided by specialists and their primary care provider—as well as to answer research questions that improve knowledge about the disorder, family adaptation, treatment benefits, and NBS program policy. All short- and long-term follow-up activities are guided by four components that we consider necessary to support families and accomplish these goals: information, support, surveillance, and treatment [42].

#### Information

Families need access to accurate and understandable information about their child’s condition, treatment options, and family implications. The conditions included in Early Check are complex, information about each is continually evolving, and primary care providers often have limited or even incorrect knowledge about them (e.g., [43]).

The Early Check website (www.earlycheck.org) serves as a primary source for information, with separate sections devoted to each disorder. We partnered with advocacy organizations for each condition to ensure accurate, current, and relevant information. Web content, in English and Spanish (the most common language after English in NC), is routinely updated with current information. Parents can submit questions that the clinical and research team use to build a “Frequently Asked Questions” section. Some information on the website is provided in a format that allows for easy printing or document sharing capabilities.

A primary goal is to ensure that print and digital content is linguistically appropriate and understandable. We follow 508 compliance regulations and apply a modified version of the Centers for Disease Control and Prevention’s (CDC) Clear Communication Index (https://www.cdc.gov/ccindex/index.html) to maximize accessibility and comprehension [44].

#### Support

Families are provided information about local and national advocacy and support groups and offered opportunities for additional genetic counseling sessions and professional support through the Early Check clinic. We are building a novel telegenetic counseling program to augment the traditional services and increase accessibility for families across the state. Telegenetic counseling has been shown to be as effective as in-person counseling [45] and will allow us to more efficiently provide high-quality genetic counseling to a larger number of families. This platform also expands our reach to families who may have limited resources, live in distant locations, or lack access to reliable transportation [46].

We will assess how parents feel about their decision to accept Early Check screening and the ways in which they use this information when interacting with their child, communicating with other family members, and finding services. Families will also provide information about their child, satisfaction with Early Check, adaptation to the condition, psychosocial well-being, and access to quality care. This information will be used to continue to build a family-centered program of support for study participants.

#### Surveillance

For SMA, FXS, and indeed most disorders to be included, Early Check will be the first time a population-based cohort has been identified at birth. Characterizing the development of these children will be of great utility in understanding short- and long-term outcomes. We will be able to identify variables associated with the timing, variation, and severity of signs and symptoms, and explore the relationship between disease phenotype and identifiable biomarkers (genetic and other). These assessments will provide invaluable natural history data and will also assure parents that someone is attending to their child’s well-being so that services or treatments can start promptly when needed.

We offer free developmental assessments at multiple timepoints during the first year of life using a battery of measures. Families can bring their children for comprehensive interdisciplinary evaluations to the Early Check clinic at UNC-CH, or we will offer a combination of home visits conducted by the research team, parent-reported measures, and tele-assessments. For families who choose to participate in the registry, we will offer ongoing, free periodic screening via parent survey, and opportunities to participate in additional studies that will involve direct assessments of the child. We will also request permission to access their child’s electronic health records to gauge health care access and utilization. Our goal is to follow children and families at 6-month intervals for at least 3 years following identification, pending the availability of funding. We will also recruit a cohort of babies who screened negative to serve as a comparison group for the true-positive babies.

#### Treatment

All children with the primary conditions identified through Early Check will likely need treatment or intervention at some point during the early childhood years—indeed, the presumed need for early treatment is a necessary prerequisite for including a condition in Early Check. The specific treatment will vary enormously depending on the disorder, the presence and severity of symptoms, and the existence of clinical trials or other studies. We attempt to balance the need for systematic data on benefits with the current realities of treatment availability, systems of clinical care that vary across the state, and parent preferences. For each disorder, we provide an initial diagnostic evaluation, followed by a parent-guided referral for treatment follow-up with both specialists and primary care providers.

The conditions included in the initial launch exemplify two of the many approaches we will likely follow. Infants with SMA, especially Type I, have a severe, life-threatening disorder for which rapid treatment is needed. Currently, the primary treatments for SMA are supportive care and nusinersen, given by intrathecal injection at several NC locations. Recently the U.S. Food and Drug Administration approved a gene therapy for SMA, Zolgensma. Parents are given this information so that they can make an informed decision about where to go for treatment and follow-up care. As other treatment options and/or clinical trials become available, we will inform parents of these options and support them and their primary care providers or specialists in making decisions about treatments and/or clinical trial participation.

In contrast, infants with FXS are at high risk for a developmental disability, but currently no medical treatment exists for the early years of this condition. The primary treatment option is referral to community-based early intervention (EI) services, which include a coordinator who monitors child development and helps families connect with appropriate service providers (e.g., developmental specialists, speech/language pathologists, physical/occupational therapists) if/when the child becomes eligible for those services. We have partnered with the NC EI services program to provide information about Early Check to providers across the state, and are conducting a survey to understand their information and support needs for serving these children and families.

In addition, we will offer an enhanced EI program to all families with an infant with FXS and will test the benefits of this program. The enhanced program will follow an established parent-mediated intervention model that has been shown to improve outcomes in infants at risk for developmental delays such as autism [47]. The intervention focuses on promoting parent sensitivity and ability to observe, understand, and address their child’s unique needs. Our goal is to determine whether an intensive early intervention program, initiated before delays become apparent, can alter developmental trajectories or reduce the likelihood or severity of secondary conditions such as anxiety, hyperarousal, or behavior problems [47].

### Evaluating outcomes

Early Check is intended to be a flexible research infrastructure that meets multiple demands for data relevant to NBS program policy. To build a successful program, we conduct formative evaluation (to develop project components), process evaluation (to monitor implementation), and outcome evaluation (to determine program success).

#### Formative evaluation

Formative evaluation informed the development of each Early Check component. For example, in developing outreach, recruitment and permissions materials, we use both qualitative and quantitative methods (e.g., interviews, focus groups, surveys, on-line experiments) to test alternative ways to create awareness, encourage parents to visit the website to consider enrollment, describe program features, characterize the disorders being screened, and help families make an informed decision about study participation. Tools and metrics such as reading level and plain language analyses help ensure that materials are as clear and accessible as possible. Likewise, the laboratory team evaluates alternative screening methods and conducts validation procedures using anonymized samples and compares them with reference samples to ensure that we identify babies with a high degree of accuracy.

#### Process evaluation

Process evaluation monitors implementation to determine if the program is working as intended. Each component of Early Check has a manual of standard operating procedures and a quality assurance plan. Data are gathered daily to assess progress towards goals and identify any gaps in procedures or other issues that need to be addressed. For example, the outreach and permissions teams document variables such as visits to the website and number of permissions by hospital. These data are used to evaluate the effectiveness of individual methods (e.g., letter, reminder postcard, social media posts, notifications through patient portals, materials in clinics, information-sharing in prenatal classes) in increasing visits to the website and study enrollment, as well as the best combination to maximize study participation. Of special interest will be the assessment of variation in participation as a function of geography, ethnicity, or low-income status. Through our LIMS system we document all laboratory activities and maintain a record of every step in the process, from accessing specimens through screening and conclusive actions (referral for diagnostic confirmation of screen positive cases or informing parents about screen negative findings). Routine assessment and monitoring of quality control samples with patient specimens allow for ongoing evaluation of test method performance characteristics and help ensure the accuracy of test results. The follow-up teams have tracking management systems to document each step after screening, including initial reports of screening results, referral of screen-positive cases for diagnostic confirmation, timely provision of counseling, and referral to treatment. The Early Check management team and task leads routinely review these data so that any necessary changes can be made in protocols and procedures.

#### Outcome evaluation

Early Check is designed to provide a mechanism to answer specific research questions, some of which cover the entire program and others of which are individualized according to the state of science and knowledge for each disorder screened. Six broad outcome domains are central to our work:

- *Study recruitment*. A primary goal of Early Check is to identify effective strategies for informing and enrolling families. We define success as proven ability to use a combination of “virtual” recruitment strategies that reach almost every family in the state at lower cost per study enrollee than would be required by 1:1 in-person models, while achieving adequate representation across varying ethnic groups and socio-economic levels. Building an evidence-based and cost-effective recruitment model will allow us to confidently predict enrollment rates for future studies and determine the likelihood of identifying enough babies within a defined period of time to answer condition-specific research questions.
- *System of services*. A fundamental tenet of NBS is that screening should only be done if services can be reasonably provided. Each disorder in Early Check will require building a statewide network of services to serve children with that condition. Here we define success as proven ability to establish the full continuum of services for each disorder, beginning with screening and including diagnostic confirmation, counseling, and appropriate follow-up and support for families. Demonstrating what is necessary to build an adequate system of services will inform NBS program policy decisions and provide critical information for states when evaluating the cost and expertise required for screening.
- *Quality and satisfaction*. Early Check will only be successful if the families who participate believe that the program has been accessible, responsive, and beneficial. To achieve this goal, we must have accessible and sufficient information; accurate screening (minimizing false negatives and false positives); few negative psychosocial outcomes attributed to Early Check participation (e.g., increased rates of postnatal depression and parental anxiety, regret for the decision to participate); prompt, individualized, and family-centered services; and no negative impact on the state’s regular NBS program (e.g., more parents who opt out of NBS). Broad-based satisfaction by study participants will support long-term recruitment efforts and provide evidence to state public health officials that researchers can partner with state public health programs in mutually beneficial ways.
- *Child and family outcomes*. Early Check documents the extent to which families and children benefit from participation. Examples of family benefit include eliminating the diagnostic odyssey, increasing access to supportive services, and informing reproductive decisions. Examples of child benefit include earlier access to services and opportunities to participate in investigational trials of new treatments. An indirect yet important benefit to the broader community of stakeholders is the data Early Check will generate on early (pre-symptomatic) development in children and the phenotypic spectrum of involvement. Although our hope is that all children and families benefit in some way from study participation, we assess a wide range of outcomes to determine if that assumption is valid.
- *Policy impact*. Early Check is designed to provide objective data to inform policy. Accordingly, we define success as providing a definitive answer to at least one policy-relevant question for each disorder included in the screening panel. For example, we are testing the benefits of early intervention for children with FXS. If we can demonstrate a significant impact on child development and behavior, it would provide policy makers the first evidence of a non-medical, pre-symptomatic treatment. For SMA, Early Check could provide important evidence about the early development of children with less severe forms of the condition and the benefits and challenges posed by earlier identification.
- *Sustainability*. Our long-term goal for Early Check is an infrastructure that will be self-sustaining through the benefits it can provide to researchers, commercial entities, and advocacy groups. We define short-term success as our ability to secure support to expand Early Check to at least one additional condition during the initial funding period, demonstrating how the program could support other researchers or groups to answer key questions in the future. Ultimately Early Check will likely constitute a more expanded panel of disorders that offers a choice to families and will inform policy decisions.

## Discussion

Patient advocacy groups, health professionals, and biomedical researchers have long lamented the well-documented lag between basic science discoveries and the eventual application of those discoveries to public health [48–50]. Although the “translational continuum” has been conceptualized in different ways [50–54], three fundamental tasks must be accomplished: (1) core disease mechanisms and pathways need to be fully understood; (2) treatment options must undergo rigorous testing in both animal models and human populations to determine safety and efficacy; and (3) clinicians and the public health system need to be ready and able to implement new treatments when they are available.

NBS occupies a unique space at the intersection of translational science and public health. As the only truly population-based public health program in the United States, NBS offers the promise of making successful applications of translational medicine available to every infant with a rare disorder that is difficult to diagnose clinically but for which there is strong evidence that outcomes can substantially improve with presymptomatic treatment. With this promise, however, comes a special obligation to ensure that the conditions included in NBS are justified. Evidence-based data, rather than conjecture, are essential to inform NBS program policy on the nature and consequences of a disorder, the accuracy of testing for it, and the harms and benefits of early diagnosis [55].

Unfortunately, standard NBS program policy decisions are often made with data that are inaccurate or incomplete, a scenario that becomes increasingly likely when disorders are rare (and not fully understood), advocates are vocal, promising (but not fully tested) treatments are emerging, and technology makes NBS more feasible [3], sometimes with unfortunate results [56]. The health of the public needs, and patient advocates deserve, a way to collect important data in a timely fashion, so that policy decisions are based on the best available evidence.

Early Check is designed to fill this gap by integrating large-scale systematic research into a state health program and a real-time NBS system. If we can achieve a reasonable voluntary participation rate across various ethnic groups and socioeconomic levels, we will be able to answer important questions about the disorders screened and the extent to which screening benefits children and families. The findings could have implications for other outreach and recruitment efforts, including effective statewide communication strategies for virtual recruitment, acceptability and feasibility of e-consent, uptake rates as a function of key demographic variables, accuracy and feasibility of screening tests, and family adaptation to screening information. Efforts to ensure voluntary enrollment and to improve parental understanding could inform future decisions about the role of parental permission in NBS and the viability of a “second-tier” permission-based platform of conditions that do not meet rigorous standards for NBS but are of great interest to families and still offer potential benefits.

As anticipated, we have been approached by researchers, patient advocacy groups, and industry groups who see the potential value of Early Check as a cost-effective way to identify infants with their condition of interest and gather data to inform clinical practice and policy. To achieve long-term sustainability, we are building a collaborative engagement model with a wide range of stakeholders and establishing a replicable and sustainable infrastructure for research and implementation that is evidence-based and can be made available to other investigative teams. Ultimately, we hope to more rapidly advance understanding of diseases and treatments, reduce the length of time it takes to include appropriate conditions in standard NBS panels, and accelerate future research on new conditions, including clinical trials for new investigational treatments.

## Data Availability

Not applicable.

## Abbreviations

BAA: Business Associate Agreement
CDC: Centers for Disease Control and Prevention
CLIA: Clinical Laboratory Improvement Amendments
ddPCR: droplet digital PCR
EI: Early intervention
FIPS: Federal Information Process Standard
FXS: fragile X syndrome
HIPAA: Health Insurance Portability and Accountability Act
IRB: Institutional Review Board
LIMS: Laboratory information management system
NBS: Newborn screening
NC: North Carolina
NC-DPH: North Carolina Division of Public Health
NCSLPH: North Carolina State Laboratory of Public Health
PCR: Polymerase chain reaction
qPCR: quantitative polymerase chain reaction
SMA: Spinal muscular atrophy
UNC-CH: University of North Carolina at Chapel Hill
